# The Use of Infrared Thermography for the Monitoring of Udder Teat Stress Caused by Milking Machines

**DOI:** 10.3390/ani9060384

**Published:** 2019-06-22

**Authors:** Francesco Maria Tangorra, Veronica Redaelli, Fabio Luzi, Mauro Zaninelli

**Affiliations:** 1 Department of Health, Animal Science and Food Safety (VESPA), Università degli Studi di Milano, Via Celoria 10, 20133 Milan, Italy; francesco.tangorra@unimi.it; 2 Department of Veterinary Medicine, Università degli Studi di Milano, Via Celoria 10, 20133 Milan, Italy; veronica.redaelli@unimi.it (V.R.); fabio.luzi@unimi.it (F.L.); 3 Department of Human Science and Quality of Life Promotion, Università Telematica San Raffaele Roma, Via di Val Cannuta 247, 00166 Rome, Italy

**Keywords:** dairy cow, milking machine, teat status, short-term stress, infrared thermography, imaging analysis

## Abstract

**Simple Summary:**

The aim of this study was to test the use of infrared thermography as a possible tool for detecting short-term stress, of cow udder teat, caused by milking procedures. Thermographic images were collected and evaluated to calculate the values of two indicators: the average and the maximum skin surface temperatures at the base, center, and tip of each teat. Obtained results confirmed a relationship between the two indicators (T_avg_, T_max_) and the level of teat stress generally evaluated by visual observation of its color. Nevertheless, the low accuracy reached by the two indicators does not seem to justify the development of an ad hoc infrared device for the monitoring of cow udder teat stress.

**Abstract:**

The aim of this study was to test infrared thermography (IRT) as a possible tool for scoring teat color changes after cluster removal; thus, indirectly, to classify the short-term stress of teats caused by milking machines. Thermographic images (*n* = 137) from three farms were collected and evaluated to calculate the average and maximum skin surface temperatures (SSTs) at the base, center, and tip of each teat (T_avg,B_, T_avg,C_, T_avg,T_, T_max,B_, T_max,C_, and T_max,T_). Obtained results confirmed a significant relationship between the indicators T_avg_, T_max_ and the levels of teat color change (level one: pink-colored teat; level two: red-colored teat; level three: blue or purple-colored teat). Nevertheless, when a teat was considered to be stressed because its scoring fell in level 3 of the color-change scale used, sensitivity and specificity in the classification of the teat status ranged respectively between 45.6% and 54.3%, and 54.4% and 59.2%, for the indicators T_avg_; and 56.5% and 60.9%, and 59.7% and 61.8%, for the indicators T_max_. When a teat was considered stressed because its scoring fell between the levels 2 and 3 of the scale adopted, sensitivity and specificity were between 49.0% and 55.8%, and 58.3% and 61.8%, for the indicators T_avg_; and 55.8% and 59.9%, and 60.6% and 61.4%, for the indicators T_max_. As a consequence, the low values of sensitivity and specificity do not seem to justify the development of an ad hoc infrared device for the monitoring of udder teat stress. Nonetheless, this technology can be a viable solution for a preliminary evaluation of the mechanical stress of teats if a milking system would be equipped with an infrared sensor already in place for other purposes (e.g., the monitoring of udder health status).

## 1. Introduction

Faults in milking machines and in milking management are the main causes of short-term effects on teats, such as discoloration after the cluster removal. Some teats can become red, either at the end or over the entire teat. Others can result in reddening within 30–60 s after the cluster removal. In extreme cases, teats can appear or can become bluish or purple-colored, indicating a cyanosis [1,2,3,4]. Different factors of milking machines can be responsible for changes in teat color. Among these factors, the following can be included: a high milking vacuum [5]; a faulty pulsation caused by a short D-phase [6,7]; a wide bore, aged and high tensioned liners [2,3,8]; a large mouthpiece chamber; and a small lip diameter mouthpiece [4]. In addition, teat color changes can be exacerbated by milking management faults, such as over-milking and mismatching between the type of liner used and the mean teat size of the herd [4].

When one or more of the aforementioned faults are not quickly detected, a mechanical stress of teats is provoked. As a long-term result, the incidence of mastitis can potentially increase, causing a reduction of milk yield and quality, an increase of costs for veterinary services and medicine, a major risk of culling, and an overall reduction of farm profitability and animal welfare.

Usually, to evaluate the color change of a teat, a visual assessment is carried out. In the milking parlor, within 30–60 s after cluster removal, a score, ranging from one to three, can be assigned to each teat. A score of one refers to a normal pink-colored teat; scores of two and three indicate that part of the teat, or its entire surface, appears respectively red or blue/purple [3,9]. However, this method of evaluation is time consuming, especially on a farm with a large quantity of cows. Furthermore, it may be subjective because of a) different lighting conditions (also in the same milking parlor) and/or b) the sensitivity of the evaluator. Additionally, black-pigmented teats cannot be scored using a color scale.

Teat color changes, caused by an incorrect setting of the milking machine (with unsuitable parameters and/or components) could be associated with a change of blood circulation and fluid retention of the teat tissue [10,11]. This effect, when it occurs, can be of the cause of an increase in teat skin surface temperature (SST), as observed in several studies [12,13,14,15]. Consequently, it should be detected, and highlighted, by infrared thermography (IRT). The physical principle at the base of IRT is explained by the laws of Planck, Wien, and Stefan–Boltzmann [16]. Each body that has a temperature higher than absolute zero emits an electromagnetic radiation in the infrared spectrum. Using a mathematical formalization/equation, the energy emitted by the body surface, the wavelength of the radiation, and the temperature of the body can be calculated. Through a dedicated sensor array, infrared radiation can be detected and used to build a thermographic image where the intensity or the color of each pixel is proportional to the corresponding temperature of the surface observed [17,18]. Many researchers have already shown the potential use of this technology in the evaluation of milking processes. For example, the working quality of different milking equipment and their influence on teat conditions has been investigated utilizing an IRT [15,19,20] to measure the SST of teats, before and after milking. Nevertheless, based on our knowledge, its potential use to classify the color changes of a teat after the milking process has never been evaluated.

Therefore, the aim of the present study was to determine if IRT could substitute the visual observation of the udder teats and, thus, allow the scoring of color changes after the cluster removal. This could enable indirect evaluation, in an objective and contact-less way, of the effects on the udder teats caused by the milking machine. As a consequence, it could permit the analysis of the correctness of the milking machine settings and/or of the milking system components on a single cow or on all farm animals.

## 2. Materials and Methods

### 2.1. Animals and Farms

A total of 137 lactating Holstein Friesian were included in this study, carried out from April to May 2018. Cows included in the experimental group were from three Northern Italian farms. Experimental animals (farm 1: *N* = 69; farm 2: *N* = 57; farm 3: *N* = 11) were balanced for number of lactations and days in milk, and did not show any sign of clinical mastitis.

All cows were reared under loose housing conditions in free-stall barns and were milked conventionally twice a day at farms 1 and 2, and automatically at farm 3. Two 2 × 12 stall herringbone milking parlors with a working vacuum level of 40 and 42 kPa were in operation respectively at farm 1 and 2, while at farm 3, an automatic milking system (AMS), single box, with a working vacuum level of 41 kPa, was active. A pulsation rate of 60 cycle/min and a pulsation ratio of 60:40 were set in all milking systems.

Animals were fed with a total mixed ration twice a day on each farm (08:00–09:00 a.m. and 17:00–18:00 p.m.) and had free access to water. Moreover, cows milked by the AMS received 1–4 kg of concentrate feed based on daily milk yield.

Ethical approval was not necessary because the experiment was designed in order to collect images in commercial farms during milking operations, without disturbing in any way the animals’ behavior or affecting the normal milking routine. Udder temperatures were acquired by a commercial infrared camera in a contactless way. The infrared camera used a passive sensor that did not emit any kind of radiation that would be potentially dangerous for animals’ health. Udder teat evaluations were carried out only by visual observations of digital pictures collected by a commercial digital camera without any contact with animals.

### 2.2. Thermographic Images Collection

Thermographic images were collected within 30–60 s after the cluster removal, keeping in mind a possible future automation of the use of the SST as a monitoring indicator of teat stress caused by the milking machine. Thermographic images were acquired through a commercial infrared camera (Thermo GEAR-G120 EX- Nippon Avionics Co, Tokyo, Japan), with an uncooled detector focal plane array (microbolometer), a resolution of 320 × 240 pixels, an accuracy of ± 2 °C, a sensitivity of 0.04 °C (at 30 °C), and sizes of 21.2 cm × 7.5 cm × 13.8 cm.

Before acquiring thermographic images, measures of temperature and relative humidity of the milking parlor were recorded. During the experimental period, these ambient parameters ranged from 15 to 25 °C (with a mean value of 21 °C) and from 67% to 78% (with a mean value of 71%). Positioning the thermal imaging camera at the center of the milking parlor and at animal height, the background radiation was determined by a dedicated feature of the camera. All ambient parameters measured were used to allow internal compensation (i.e., calibration) by thermal imaging camera algorithms. Furthermore, an auto-calibration function of the infrared camera was left active. This allowed for the ability to make subsequence (and automatic) calibrations of the thermal imaging camera when it was necessary. All these settings allowed for the ability to collect thermographic images not affected by the maximum “theoretical” error declared by the thermal imaging camera manufacturer (i.e., ± 2 °C). An emissivity value of 0.98, selected in accordance with other published studies carried out on udder SST [21,22,23,24,25,26,27], was used to complete the settings of the thermal imaging camera. Prior to obtaining each thermographic image, the infrared camera operator ensured optimum image focus.

Thermographic images were collected by positioning the infrared camera at udder level, at a distance of circa 0.5 m [26,28,29,30,31] from the lateral part of each udder, in accordance with the frame of the milking parlor. One thermographic image was acquired for each cow and included a fore and hind udder quarter. During the acquisition of thermographic images, multiple images were collected in order to guarantee (for each udder) at least one clear image to use for the image analysis that followed.

**Figure 1:**
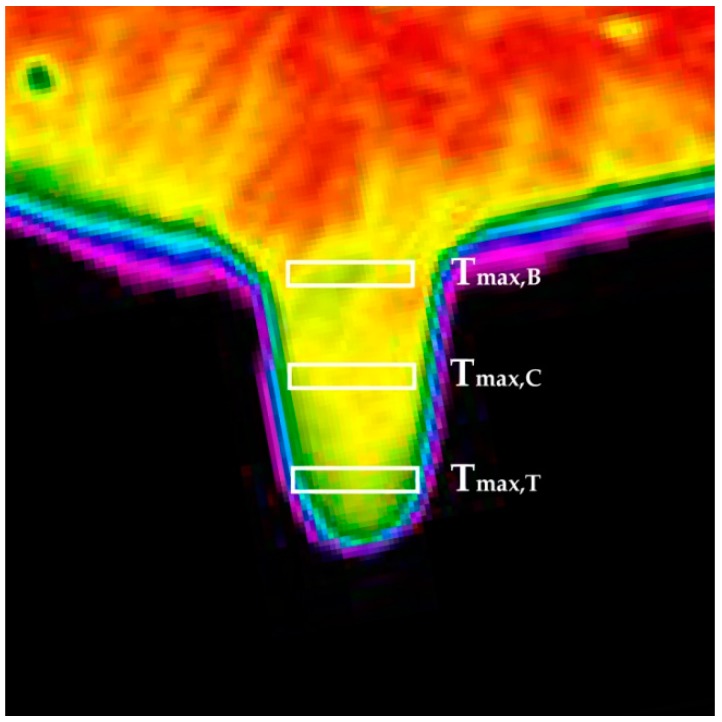
An example of an image acquired and elaboration performed in the study carried out. In detail, it is a reported example of a thermographic image of a teat and the areas of rectangular shape considered for the calculation of the six teat udder skin temperatures (i.e., T_avg,B_, T_avg,C_, T_avg,T_, T_max,B_, T_max,C_, and T_max,T_). As shown, these three areas were centered at the base, at the center, and on the tip of each udder teat, and the values of the indicators T_avg/max,B_, T_avg/max,C_, and T_avg/max,T_ were the average and maximum surface temperatures measured in these three areas, respectively.

All thermographic images were analyzed by a professional software tool (InfRec Analyzer NS9500 Lite - version 2.7A, Nippon Avionics Co, Tokyo, Japan). For each teat that was visible in the thermographic image, six teat SSTs were calculated (T_avg,B_, T_avg,C_, T_avg,T_, T_max,B_, T_max,C_, and T_max,T_). These teat SSTs were the average and maximum temperatures measured in three areas of a rectangular shape of 5 pixels in length and 25 pixels in width. In each teat, these rectangles were manually positioned—one at the base (to calculate the T_avg/max,B_ value), one at the center (for the T_avg/max,C_ value), and the last one at one centimeter above the teat tip (to calculate the parameter T_avg/max,T_, shown in Figure 1).

### 2.3. Digital Pictures Collection and Udder Teat Evaluations

After the collection of each thermographic image, a digital picture was also captured for each udder using a commercial digital camera (Panasonic, Lumix DMC-FS40, Kadoma, Osaka, Japan). The camera had a resolution of 4320 × 3240 pixels, a focal length of 24 mm to 120 mm, a diaphragm aperture of 2.5 to 6.4, while its sizes were 95 mm × 55 mm × 20 mm. Before acquiring each sequence of digital pictures, a manual white balance was performed in order to avoid any possible color shift caused by different and mixed ambient lights. Furthermore, “shooting mode” was set up on “automatic” in order to reach, in each acquired image, the correct focus and exposure.

Digital photographs were collected by positioning the camera at udder level—at a distance of circa 0.5 m from the lateral part of each udder—coherently with the frame of the milking parlor. At least one digital picture, including a fore and hind udder quarter, was acquired for each cow.

**Figure 2:**
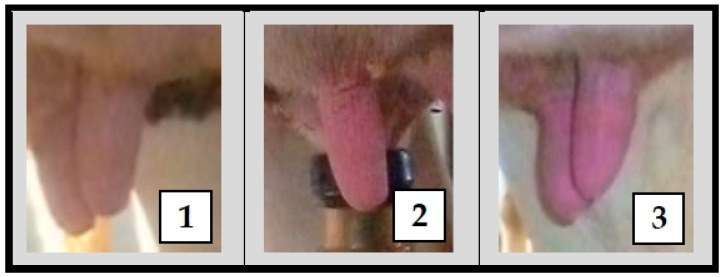
An example of classification of teats after the cluster removal. The classification was based on the color change of the teat and it used a color-change scale that had the following meanings: (**1**) for a normal pink-colored teat; (**2**) when the whole teat, or a part of it, was red-colored; and (**3**) if the whole teat, or a part of it, was blue or purple-colored.

Digital pictures were used in the classification procedure of teat stress caused by the milking machine. The classification of each teat correlated with a scale that ranged from one to three, based on color change of the teat [32]. In particular, the values of the scale had the following meanings: (1) for a normal pink-colored teat; (2) indicated that the whole teat, or a part of it, was red-colored; and (3) specified that the whole teat, or a part of it, was blue or purple-colored (Figure 2).

### 2.4. Statistical Analysis

Data collected in the study were investigated through statistical analysis performed using the “R” software tool (version 3.5.0, 2018) [33]. Associations between the indicators T_avg_ and T_max_ (i.e., T_avg,B_, T_avg,C_, T_avg,T_, T_max,B_, T_max,C_, and T_max,T_) and each level of the teat color-change scale were evaluated using a specific linear mixed-effects model (procedure *lme* [34] of the package nlme “Linear and Nonlinear Mixed Effects Models”, version 3.1-137). The linear model fitted was as follows:

(1) $$Y_{ijk}=\mu +TC_{i}+q_{j\left(k\right)}+c_{K}+e_{ijk},$$

where Y is the teat SST (for each teat area investigated); TC_i_ is the effect of the teat colour change after the performing of a milking (i = 1–3, 1 = pink-colored teat, 2 = red-colored teat, 3 = blue or purple-colored teat); q_j(k)_ is the random effect of the teat (j = 1–2; 1 = fore teat, 2 = hind teat) nested in the cow (k = 1–137) [35] ; c_k_ is the random effect of the cow (k = 1–137); and e_ijk_ is the residual error. Obtained results were considered significant when *p*-values were lower than 0.05. 

In the statistical elaborations that followed, the ability of the indicators T_avg_ (T_avg,B_, T_avg,C_, T_avg,T_) and T_max_ (T_max,B_, T_max,C_, T_max,T_) to detect a possible case of teat stress caused by the milking machine were evaluated. When each T_avg_ or T_max_ overcame a specific threshold, a case of teat stress was supposed. As a consequence, the result of a possible statistical test was set up as “positive”. Positive results were compared with the teat classifications performed on the basis of the possible color changes induced by the milkings. The results of all these comparisons were classified as the following: true positive (TP), when the statistical test was able to detect a real case of mechanical stress of the teat; false positive (FP), when the statistical test supposed a case of mechanical stress of the teat while the teat was not truly being stressed; true negative (TN), when the statistical test correctly detected a case of the teat not mechanically stressed; and false negative (FN), when a teat mechanically stressed was not identified by the statistical test. After the classification of all comparisons, the performances of the statistical tests based on the evaluations of the indicators T_avg_ and T_max_ were calculated as either sensitivity or specificity, in accordance with the following formulas:

(2) $$\mathrm{Sensitivity}\;\left[\%\right]=\frac{\mathrm{TP}}{\mathrm{FN}+\mathrm{TP}},$$

(3) $$\mathrm{Specificity}\;\left[\%\right]=\frac{\mathrm{TN}}{\mathrm{FP}+\mathrm{TN}}.$$

As expected, statistical tests gave different couples of sensitivity and specificity for each possible threshold used to evaluate the indicators T_avg_ and T_max_. Thus, receiver operating characteristic curves (ROC) were built using the procedures “*prediction*” and “*performance*” of the package “*ROCR*” (version 1.0.7 [36]). Analyzing these curves, specific cutoffs were selected and the corresponding couples of sensitivity and specificity were identified as the final performance reached by the indicators. Furthermore, the areas under the curves (AUC) were measured to compare the global performance reached by each indicator in the detection of a possible case of mechanical stress of a teat, caused by the milking machine.

All statistical analyses were repeated considering the two criteria of classification of the teat status. The first considered a teat as “mechanically stressed” when the scoring of the teat fell in the level “three” of the color change scale (i.e., when the teat after milking was recognized to be blue or purple-colored). The second criterion, instead, considered a more restrictive approach. It considered a teat as “mechanically stressed” when the scoring of the teat fell within a range of the color change scale from “two” to “three” (i.e., when the teat after milking was recognized to be red-, blue-, or purple-colored).

## 3. Result

The first step of statistical analysis investigated the relationships between the indicators T_avg_ and T_max_ (i.e., T_avg,B_, T_avg,C_, T_avg,T_, T_max,B_, T_max,C_, and T_max,T_) and each level of the udder teat color scale. The mean values of the indicators and their significances for each level of the color-change scale adopted to evaluate the teats are reported in Table 1.

**Figure 3:**
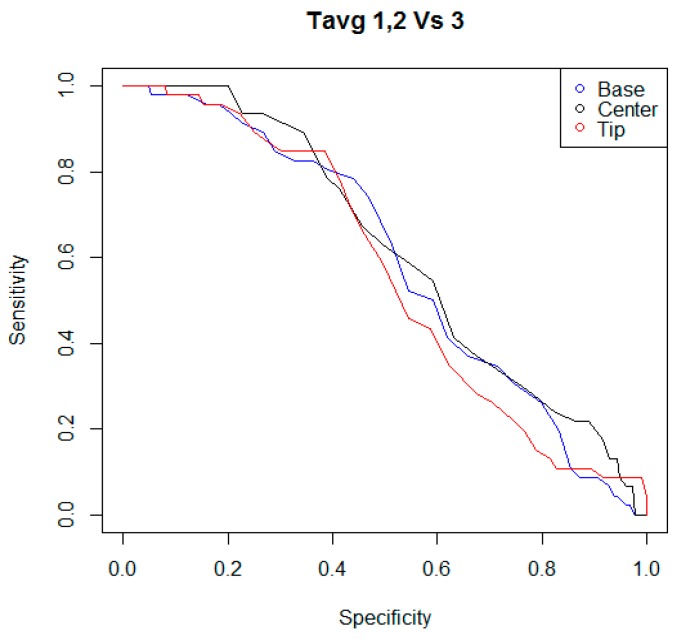
Receiver operating characteristic curves (ROC) curves of the statistical tests that were built considering the performances of the indicators T_avg,B_, T_avg,C_, and T_avg,T_, and different possible cutoff levels. The indicators T_avg_ were the average surface skin temperatures measured by the thermal imaging camera in areas of rectangular shape, positioned at the base, at the center, and at the tip of each teat. Teat status was evaluated while considering digital pictures acquired within 30–60 s after the cluster removal. Through these digital pictures, color changes of teats, caused by a possible mechanical stress, were evaluated by a researcher adopting a scale that ranged between one to three. When teat scoring fell in the level “three” of the color change scale, the teat was considered as “mechanically stressed”.

**Figure 4:**
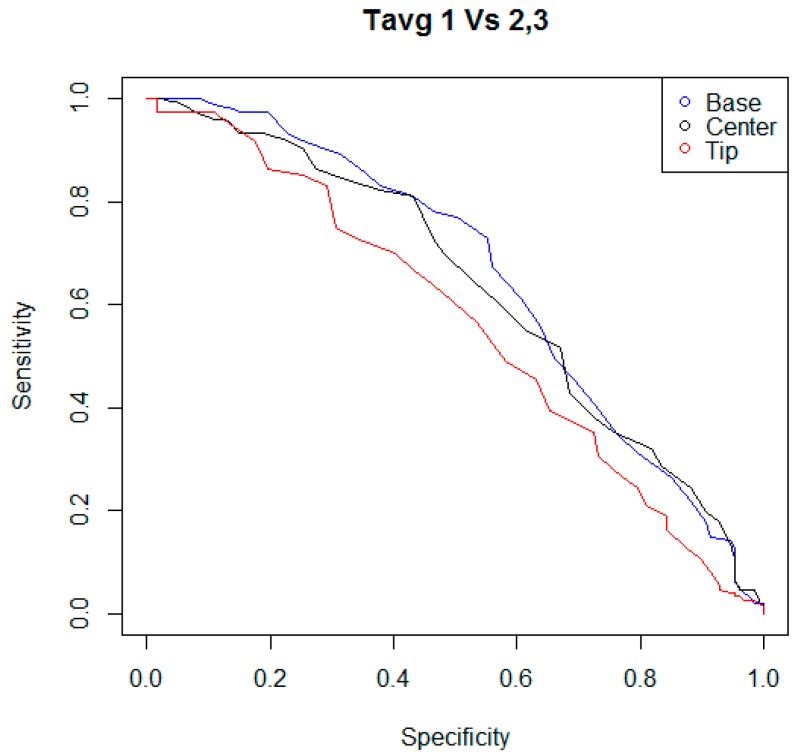
ROC curves of the statistical tests that were built considering the performances of the indicators T_avg,B_, T_avg,C_, and T_avg,T_, and different possible cutoff levels. Teat status was considered as “mechanically stressed” when its scoring fell in the level “two” or “three” of the color change scale adopted.

**Figure 5:**
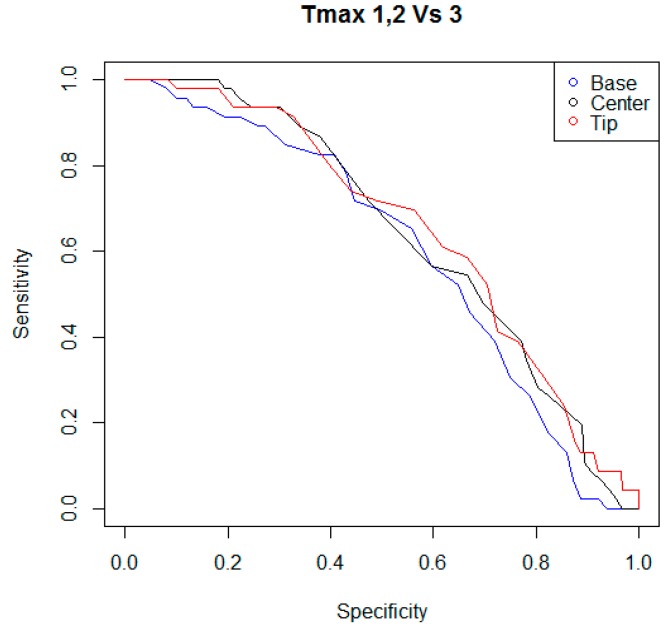
ROC curves of the statistical tests that were built considering the performances of the indicators T_max,B_, T_max,C_, and T_max,T_, and different possible cutoff levels. The indicators T_max_ were the maximum surface skin temperature measured by the thermal imaging camera in areas of rectangular shape, positioned at the base, at the center, and at the tip of each teat. Teat status was considered as “mechanically stressed” when its scoring fell in the level “three” of the color change scale.

**Figure 6:**
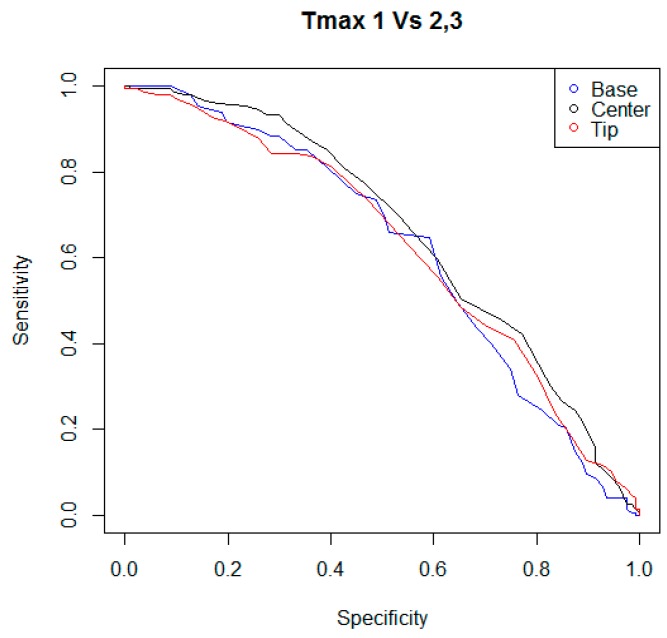
ROC curves of the statistical tests that were built considering the performances of the indicators T_max,B_, T_max,C_, and T_max,T_, and different possible cutoff levels. Teat status was considered as “mechanically stressed” when its scoring fell in the level “two” or “three” of the color change scale adopted.

In the following step of statistical analysis, the detection performances of the indicators T_avg_ and T_max_ were investigated using two criteria to classify the teat status. For each kind of indicator, and for each criterion of teat status classification, a ROC curve was calculated, considering the couples of sensitivity and specificity of the statistical test when different possible cutoff levels were adopted. In Figure 3, Figure 4, Figure 5 and Figure 6, all ROC curves obtained are reported.

In a final phase of statistical analysis, the AUCs and the final cutoff levels were determined for each ROC curve calculated. Final cutoff levels were identified considering the points in the curves closer to the best theoretical results (i.e., the point in the graph in the upper right corner equal to a sensitivity and specificity of 100%). Results obtained are reported in Table 2. Also, means and standard error values of the main indicators were investigated, and were calculated for each criterion adopted to classify the teat status. Results obtained are shown in Table 3.

## 4. Discussion

The relationship between the levels of color changes of teats—used to evaluate the short-term effects of milking machines—and the T_avg_ and T_max_ values—calculated on thermographic images collected—were shown to be significant (with the exception of the indicator T_avg,T_ that showed a *p*-value equal to 0.06). For each indicator evaluated (T_avg,B_, T_avg,C_, T_avg,T_, T_max,B_, T_max,C_, and T_max,T_), surface skin temperatures of purple- and red-colored teats were higher when compared to pink-colored teats. This suggests that an increase of the teat SST, at the base of the teat as well as at the center or at the tip, can be observed when a mechanical stress of the teat occurs. This result is in accordance with previous experiments. Several thermographic measurements of teats, in fact, showed that milking may cause traumatization of certain zones of the teat [37,38] and increase its SST [13,15,39,40,41,42]. According to Paulrud et al. [14], this effect can be explained considering that a decrease in the teat tone during milking causes the opening of arterioles and arterio-venous anastomoses, with a marked increase in bloodflow and a higher convective heat loss from the skin.

In our study, mean values of the teat SSTs grew differently at the base, the center, and the tip of the teat, and in correlation with a change of teat color (from pink- to red-, blue- or purple-colored). This result confirms that milking can affect teat temperature differently depending on the area of the teat [14]. A similar trend was observed also by Alejandro et al. [31]. Studying the effect of the milking machine on teat tissue changes in Murciano-Granadina goats, they found that milking causes a more significant increase of the mean temperature at the teat tip than at a growing distance from the teat end. On the contrary, our result differs from that of Paulrud et al. [14]. In a study aimed to evaluate by infrared thermography the effects of liner characteristics and over-milking on teat temperatures, the authors found that, during milking, mid-teat temperature increased greatly, while both base and teat tip temperature increased less, or slightly decreased, depending on the liner used. However, this conflicting result might be explained by considering the technical parameters of the milking systems and the liner designs adopted during the experiments were different in our study.

In our investigation, the indicators T_avg_ and T_max_ were also evaluated to determine the presence of a mechanical stress on the teat caused by the milking machine. Two criteria were adopted to classify teat status. The first criterion considered a teat as mechanically stressed when the teat scoring fell in the level three of the color-change scale. The second criterion considered a teat as mechanically stressed when the teat scoring fell in a range of levels, from two to three, of the color-change scale used in the study. For the indicators T_avg_, the performances of the tests ranged between 45.6% to 54.3% for sensitivity and 54.4% to 59.2% for specificity, when the first criterion was considered to classify the teats status; or 49.0% to 55.8% for sensitivity and 58.3% to 61.8% for specificity when the second criterion was adopted for the classification of teats. For the indicators T_max_, the performances of the tests ranged between 56.5% to 60.9% for sensitivity and 59.7% to 61.8% for specificity when the first criterion was evaluated in the classification procedure of teats; or 55.8% to 59.9% for sensitivity and 60.6% to 61.4% for specificity when the second criterion was used to study the status of teats. Furthermore, for each indicator T_avg_ and T_max_, and for each classification criterion considered, a ROC curve was calculated and the corresponding AUC was measured. For the indicators T_avg_, the diagnostic accuracies ranged between 0.55 to 0.61 when the first criterion was considered to classify the teats status; or 0.56 to 0.65 when the second criterion was adopted for the classification of teats. For the indicators T_max_, the diagnostic accuracies ranged between 0.60 to 0.65, when the first criterion was evaluated in the classification procedure of teats; or 0.62 to 0.66, when the second criterion was used to study the status of teats. In general, the indicators T_max_ showed better performances than the indicators T_avg_, and the best result was reached by the indicator T_max,T_ considering the first criterion to classify the teat status (sensitivity of 60.87, specificity of 61.84, and AUC of 0.657). However, the overall results of the tests evaluated do not seem to justify the development of an ad hoc IR thermographic device for the monitoring of the effects of milking on the teats. Nevertheless, the authors hypothesize that in the case that an IR sensor already equips a conventional or automatic milking system for other purposes (e.g., the monitoring of udder health status [43,44]), the same sensor could be used as a preliminary evaluation method of the mechanical stress of teats caused by the milking machine. Through a specific setting of the alarm threshold, the number of false positive cases could be limited and an automatic and continuous evaluation of teats could be implemented, enabling early detection of possible faults of the milking machine. As a result, a possible improvement in the management of the herd’s health status could be achieved.

## 5. Conclusions

The indicators T_avg_ and T_max_ showed a significant relationship with the levels of color changes used to evaluate the short-term effects of milking machine on teats, highlighting a potential use of these indicators for the detection of the teat mechanical stress. However, the low sensitivity and specificity found, considering two criteria of classification of the teat as “mechanically stressed”, seem to not justify the development of an ad hoc IR thermographic device for the monitoring of the teat status. Nevertheless, this technology might be considered for an online monitoring of teats if an IR sensor already equips a milking system for other collateral purposes. 

## Figures and Tables

**Table 1:** Values of the indicators T_avg,B_, T_avg,C_, T_avg,T_, T_max,B_, T_max,C_, and T_max,T_ for each level of the color change scale used to evaluate the teats after milking (means ± S.E.).

Indicator	Teat Color Change Scale	Mean (°C)	Standard Error	Significance
**T_avg,B_**	1	33.92	0.09	*p* < 0.01
	2	34.44	0.08	
	3	34.47	0.09	
**T_avg,C_**	1	34.54	0.08	*p* < 0.01
	2	34.90	0.08	
	3	35.07	0.10	
**T_avg,T_**	1	34.83	0.09	*p* = 0.06
	2	34.97	0.09	
	3	35.19	0.14	
**T_max,B_**	1	34.62	0.10	*p* = 0.01
	2	35.00	0.09	
	3	35.07	0.10	
**T_max,C_**	1	35.50	0.08	*p* < 0.01
	2	35.96	0.08	
	3	36.12	0.08	
**T_max,T_**	1	36.39	0.08	*p* < 0.01
	2	36.66	0.08	
	3	36.97	0.10	

**Table 2:** Final performances of the statistical tests based on the evaluation of the indicators T_avg,B_, T_avg,C_, T_avg,T_, T_max,B_, T_max,C_, and T_max,T_ are shown. In the table, areas under the curves (AUC), sensitivity, specificity, and the corresponding cutoff level are reported for each indicator and criterion of reclassification of the teat status. The values reported in the table were calculated through a customized function developed for the “R” statistical software tool.

Color Change Scale Value Used to Classify a Teat Stressed by the Milking Machine	Indicator	AUC (Area)	Sensitivity (%)	Specificity (%)	Cutoff Level (°C)
3	T_avg,B_	0.586	52.17	54.38	34.4
	T_avg,C_	0.612	54.35	59.21	35.0
	T_avg,T_	0.559	45.65	54.39	35.1
	T_max,B_	0.600	56.52	59.65	35.1
	T_max,C_	0.642	56.52	59.65	36.0
	T_max,T_	0.657	60.87	61.84	36.8
2 and 3	T_avg,B_	0.651	55.78	61.78	34.4
	T_avg,C_	0.630	55.10	61.42	34.9
	T_avg,T_	0.560	48,98	58.27	35.1
	T_max,B_	0.617	55.78	61.42	35.0
	T_max,C_	0.652	59.86	60.63	35.9
	T_max,T_	0.623	55.78	60.63	36.7

**Table 3:** Descriptive statistics of the indicators investigated (T_avg,B_, T_avg,C_, T_avg,T_, T_max,B_, T_max,C_, and T_max,T_) in terms of mean and standard error values (S.E.) for each criterion adopted to classify the teat status (i.e., criterion 1: teat = “stressed” if the scoring of the teat fell in the level “three” of the color change scale adopted to classify each teat after the end of milking; criterion 2: teat = “stressed” if the scoring of the teat fell within the level “two” or “three” of the color scale change).

Color Change Scale Levels Used to Classify a Teat Stressed by the Milking Machine	Indicator	Teat Status (Not Stressed/Stressed)	Cases (*n*)	Temperatures (°C, Means ± S.E.)	Significance
3	**T_avg,B_**	not stressed	228	34.17 ± 0.06	=0.13
		stressed	46	34.46 ± 0.10	
	**T_avg,C_**	not stressed	228	34.70 ± 0.06	<0.05
		stressed	46	35.07 ± 0.10	
	**T_avg,T_**	not stressed	228	34.90 ± 0.07	=0.11
		stressed	46	35.19 ± 0.14	
	**T_max,B_**	not stressed	228	34.79 ± 0.07	=0.14
		stressed	46	35.07 ± 0.10	
	**T_max,C_**	not stressed	228	35.70 ± 0.06	=0.01
		stressed	46	36.12 ± 0.09	
	**T_max,T_**	not stressed	228	36.51 ± 0.06	<0.01
		stressed	46	36.97 ± 0.10	
2 and 3	**T_avg,B_**	not stressed	127	33.93 ± 0.07	<0.01
		stressed	147	34.46 ± 0.07	
	**T_avg,C_**	not stressed	127	34.54 ± 0.08	<0.01
		stressed	147	34.95 ± 0.07	
	**T_avg,T_**	not stressed	127	34.83 ± 0.09	=0.12
		stressed	147	35.04 ± 0.08	
	**T_max,B_**	not stressed	127	34.62 ± 0.10	=0.01
		stressed	147	35.02 ± 0.07	
	**T_max,C_**	not stressed	127	35.50 ± 0.08	<0.01
		stressed	147	36.01 ± 0.06	
	**T_max,T_**	not stressed	127	36.39 ± 0.08	<0.01
		stressed	147	36.76 ± 0.07	
